# Progress of Surgery

**Published:** 1901-05-18

**Authors:** 


### Progress of Surgery.

(*Continued from page* 101.)

OTOLOGY (*concluded*).

**Aural Exostosis.**—This subject was discussed at the January meeting of the British Laryngological Association. Messrs. Browne, Collier, and Kelson brought forward cases. Judging from the remarks made by these and other members of the society, the views of the majority seem to be as follows: that removal of the growths should be avoided unless imperatively necessary; that the cases which were more or less pedunculated, and which were true exostoses and originated from granulations, furuncles, and the like were the easiest to cure. When hyperexostosis was present, originating in inflammation of the periosteum, it was well to treat merely by removing accumulation behind the bony overgrowth, and avoid an operation which was not without danger and often did not yield a satisfactory result. A very small opening is sufficient for hearing purposes. Unbearable tinnitus might justify operation. The exostosis is usually cancellous when there has been a suppurative cause.

**Cholesteatoma of the Temporal Bone.**—H. J. Waring has contributed a paper on this subject.[6] A cholesteatoma is a tumour composed of a large mass of epithelial cells, squamous in character. The swelling is localised, generally oval-shaped, and situated in the interior of the temporal bone either in the antrum, mastoid cells, or the tympanic attic. The cells of the mucous membrane in these cavities are cubical or columnar. Cholesteatomata are primary or secondary. The first group comprises those cases in which the tumour is supposed to be derived from the epithelium of a cul-de-sac which extends from the deeper part of the external auditory canal towards the antrum and tympanic cavity. Another origin may be from a portion of surface epithelium which has become included during development of the middle ear. The second group comprises those cases which develop in association with long-continued suppuration of the middle ear and partial destruction of the tympanic membrane. Squamous-celled epithelium then grows into the tympanum through the aperture in the membrane, and the tumour grows from it. Very few of the cases belong to the primary group. Waring then gives clinical records of a case of each kind. The first case is that of a woman of twenty-two who complained of a painful swelling behind the external auricle. There had never been any ear discharge. The swelling was incised after three weeks and pus let out. A bare bone was felt. Three weeks later Waring explored the antrum. A large cavity was found, which, after firm tumour-like material had been completely removed, measured 2 inches in its long diameter and about 1¼ inch in the other. The cavity was treated by packing for several weeks, and became lined with granulations. It was rendered aseptic by frequent irrigations, and then a further operation done. The cavity was exposed and entirely filled with long thin strips of bone and cartilage taken from a kitten's bones. The external wound was closed by sutures. She left the hospital in a fortnight with the sinus healed and practically no ear discharge. She was quite well when seen two years later. This case is an example of a primary cholesteatoma. Next a case of the secondary type is detailed in which similar treatment was adopted. The operation failed to cure the condition, probably because the cavity was not rendered completely aseptic. Ballance's operation was finally performed with moderately good results. Kuhn's case of the primary type is alluded to. That surgeon, operating for a mastoid abscess, found a cavity in the temporal bone the size of a hen's egg, which was filled with a cholesteatomatous tumour. Removal of this exposed the lateral sinus and cerebellum. Waring alludes to some excellent results he has obtained in suppurative otitis by irrigating with formalin. This substance is volatile, and hence acts better than some other materials. It is suggested that in children the cavity left in the bone might be filled by moving forwards into it a partially resected piece of the mastoid process. With regard to the use of formalin L. Vascher also claims to have had good results with it in chronic otitis. He used a 1 in 20 solution. After irrigating with that he packs the auditory canal with gauze soaked in the solution, or in one of 10 per cent. strength. Otorrhœas of long standing have been controlled in this way in fifteen days. C. H. Burnett[8] says he would hesitate to use formalin in stronger solutions than 1 in 1,000 or 1 in 500 owing to the pain it produces.

**Tuberculosis of the Middle Ear.**—Seymour Oppenheimer[9] has written on this subject. In the majority of cases the infective material is carried to the ear by the Eustachian tube. Primarily the tuberculous nodule, as seen on the promontory or near the tubal opening, is red and slightly elevated, and the mucosa pale and grey from leucocyte invasion. The ossicula are frequently exfoliated, and at times even the foot-plate of the stapes gives way and infection spreads to the internal ear. Necrosis may attack the promontory and other parts of the wall of the tympanum, and spread to the canal of the facial nerve. The development is insidious, pain is conspicuous by its absence, and there is early enlargement of the periotic glands. In testing for the bacillus the discharge should be taken from the depths of the cavum tympani, and repeated examinations must be made, especially when there is in addition profuse otorrhœa from streptococcal invasion. In treatment constitutional remedies are of the greatest importance. Surgical treatment is contraindicated except where there is no other tuberculous focus elsewhere, and where the area of aural infection is limited. When operating every portion of diseased tissue should be removed, and a free open wound allowed to remain. This should be frequently packed with iodoform gauze.

**Otitic Lateral-Sinus Thrombosis.**—O. Joachim,[10] discussing the question of ligation of the jugular vein for this condition, says that other avenues of infection on one hand, and the presumption of an occluding clot in the jugular on the other, seem to argue against the necessity of always tying the vein; but the preponderance of recoveries being in the cases in which ligation has been adopted, he personally favours that procedure when a high degree of pyæmia is evident. This question has been discussed by J. Lacy Firth,[11] who records a case of septic thrombosis successfully treated without ligaturing the vein. The patient, a male aged 26, had had four rigors, malaise, headache, and vomiting during the seven days which preceded the operation. There was no tenderness or thickening of the jugular vein, nor mastoid tenderness. When a disc of bone was removed over the lateral sinus flocculent pus immediately escaped from an extra-dural collection. The outer sinus wall looked like a slough. When laid open clot was found in it of dark colour externally, but disintegrating in the centre. It was removed with a sharp spoon, and free bleeding excited from both the torcular and the jugular ends. This was easily arrested by plugging. Recovery was complicated after eight days by the formation of an abscess in the upper part of the neck communicating with the cranial cavity; in fact, an abscess of the type described by Bezold. This healed up without further operation, apparently draining through the subdural space and the trephine aperture. Firth quotes other cases of sinus thrombosis which have made good recoveries, and, in conclusion, says that it appears to him that a study of the records he has quoted goes far to show that ligature of the jugular vein in sigmoid sinus thrombosis need not, in many cases, be regarded as an essential part of the operation, and that it will usually be better practice not to ligate the vein in cases operated upon before embolic abscesses have formed, if a free flow of blood can be established from both ends of the sinus by removal of the thrombi, or if firm, healthy-looking clot is found plugging the sinus at the lower end. R. A. Wilson[12] records an interesting case of mastoid disease leading apparently to otitis media, and to fatal pyæmia without immplication of the meninges or lateral sinus. The patient was an epileptic who had slight deafness and slight mastoid pain of some five years' standing, the result of an old injury. Then he sustained a contusion of the right ear, and eight days later developed purulent otitis and died three weeks later, after having had several pyæmic joint abscesses opened. At the post-mortem the meninges and sinus were found unaltered.

**Otitic Extradural Abscess.**—James F. M'Caw[13] reports two cases of this condition. In the second case acute otitis followed influenza, and very free discharge persisted to flow from the ear for six weeks. This did not prevent the patient attending to his business, which he did up to the time he was sent to the hospital for operation. There was very slight mastoid tenderness, yet at the operation the whole mastoid was found filled with pus and very necrotic. The inner mastoid cortex had disappeared, exposing half an inch of the sigmoid sinus, which was bathed in pus and necrotic tissue. M'Caw points out that this case demonstrates the treacherous and insidious nature of the disease, and illustrates the danger in delaying treatment. This patient was attending to his business up to the very day of the operation, and it was difficult for him to understand that an operation was imperative. The dangerous character of influenza otitis is remarked upon. Knapp says:—"The grippe is a frequent cause of mastoid caries." Fortunately only one-quarter of the intracranial complications follow acute suppuration of the middle ear, while chronic otorrhœa is responsible for three-quarters.

[6] Edin. Med. Jour., Feb. 1901. [7] Vide Abs. Practitioner, Feb. 1901. [8] Amer. Year-book of Med. and Surg., 1900. [9] Vide Abs. Med. Chron., Feb. 1901. [10] Vide Abs. Med. Rec., Nov. 1900. [11] Bristol Medico-Chir. Jour., March, 1901. [12] Vide Abs. Jour. Laryng. R. and O., Dec. 1900. [13] Laryngoscope, Feb. 1901.

